# Linear IgA Bullous Dermatosis Attributable to the Use of Spironolactone: A Case Report

**DOI:** 10.7759/cureus.40690

**Published:** 2023-06-20

**Authors:** Vineetha Philip, Olushola O Ogunleye, Nneka Chukwu, Isaac Rosenblum, Susan Collins

**Affiliations:** 1 Rheumatology, Houston Methodist Hospital, Houston, USA; 2 Internal Medicine, Vassar Brothers Medical Center/Nuvance Health, Poughkeepsie, USA; 3 Dermatology, Hudson Dermatology, Fishkill, USA

**Keywords:** bullous rash, direct immunofluorescence, drug-induced vesiculobullous disease, spironolactone, iga bullous dermatosis

## Abstract

Linear IgA bullous dermatosis (LABD) is a rare, idiopathic, or drug-induced vesiculobullous disease caused by IgA autoantibodies in the basement membrane zone. An 84-year-old man was started on spironolactone two weeks before presentation for the management of hypertension and heart failure with preserved ejection fraction. He presented to our hospital for evaluation of worsening lower extremity swelling and a painful pruritic rash that started on the day preceding his presentation. On examination, he had 3+ lower extremity edema and an erythematous, painful, pruritic, bullous rash on all his extremities. He had a significantly elevated IgA level (1033 mg/dL). A lesional skin biopsy demonstrated epidermal ulceration with degenerated collagen fibers. Direct immunofluorescence of the perilesional skin showed linear IgA at the dermal-epidermal junction. The rash resolved following steroid therapy and discontinuation of spironolactone. There have been previous reports of bullous pemphigoid induced by spironolactone. To our knowledge, LABD associated with spironolactone has not previously been reported.

## Introduction

Linear IgA bullous dermatosis (LABD) is a rare, autoimmune, vesiculobullous disease with an annual incidence of 0.2 to 2.3 cases per million population. It is caused by IgA autoantibodies produced against several antigens in the basement membrane zone [1,2]. There are childhood-onset and adult-onset forms [3-5]. Childhood-onset LABD occurs between ages six months to 10 years (mean age of 4.5 years), while adult-onset LABD has two peaks of disease presentation: in post-pubertal teenagers, and above 60 years of age [3-6]. Clinical presentation of LABD in adults varies from diffuse blisters resembling bullous pemphigoid to vesicular lesions akin to herpetic disease, occasionally manifesting as a mimicker of Stevens-Johnson syndrome or toxic epidermal necrolysis [7].

Although the etiology of LABD is mostly idiopathic, LABD has been associated with certain medications, and systemic autoimmune diseases such as rheumatoid arthritis, systemic lupus erythematosus, inflammatory bowel diseases, and malignancies [3]. Common drugs implicated in LABD include vancomycin, penicillin, trimethoprim-sulfamethoxazole, angiotensin-converting enzyme inhibitors, and nonsteroidal anti-inflammatory agents [3,7]. The exact mechanism by which drugs induce the immune system of a susceptible individual to produce IgA antibodies against the basement membrane in LABD is still unknown [8,9]. Some drugs may act as haptens, complexing with dermal/epidermal proteins and eliciting an autoimmune response [9]. Upon review of the current literature, there have been reports of spironolactone-associated bullous pemphigoid [10], but spironolactone has not previously been implicated in the etiology of LABD. We present a case of LABD likely due to spironolactone use.

## Case presentation

**Figure 1:**
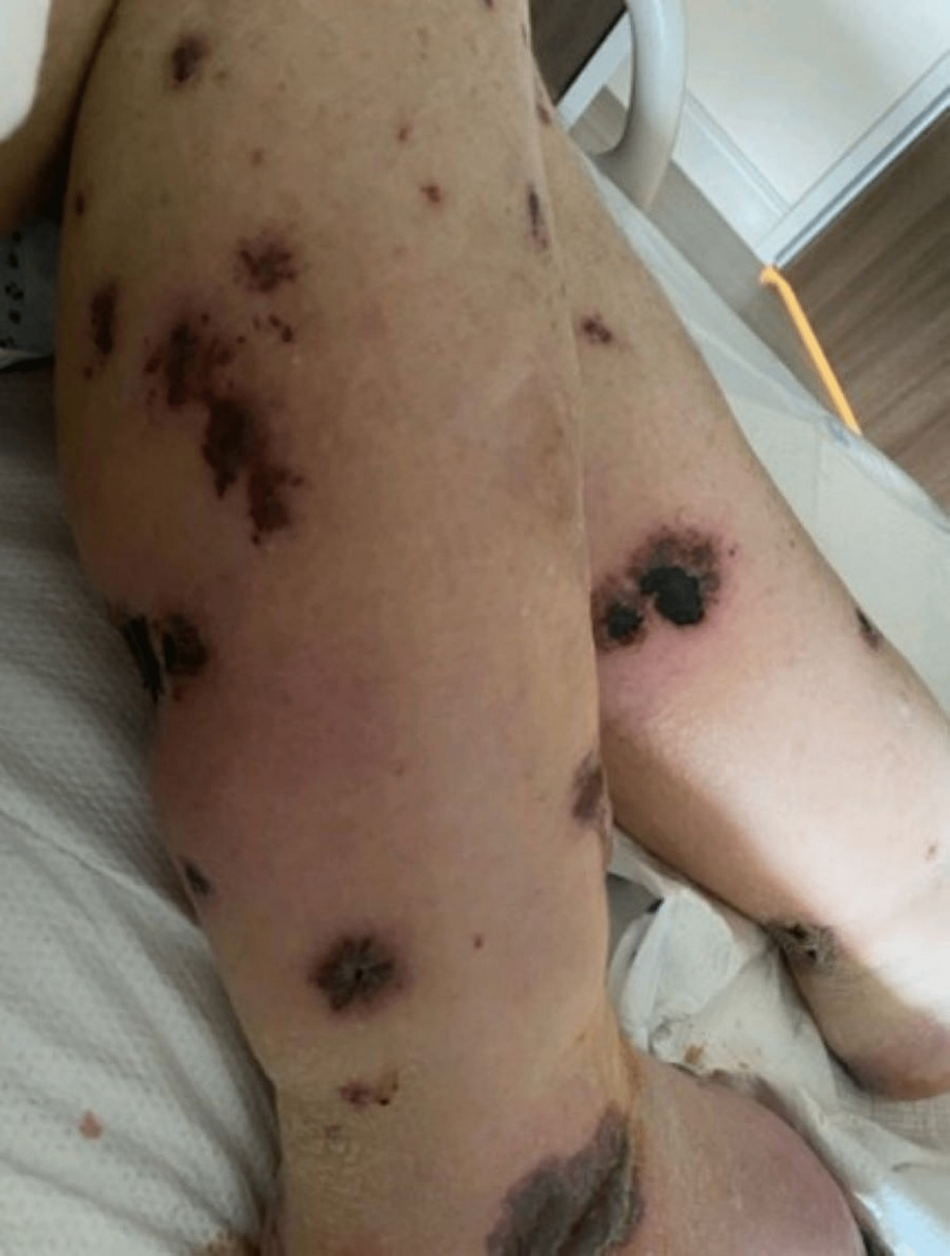
Rash on lower extremities

**Figure 2:**
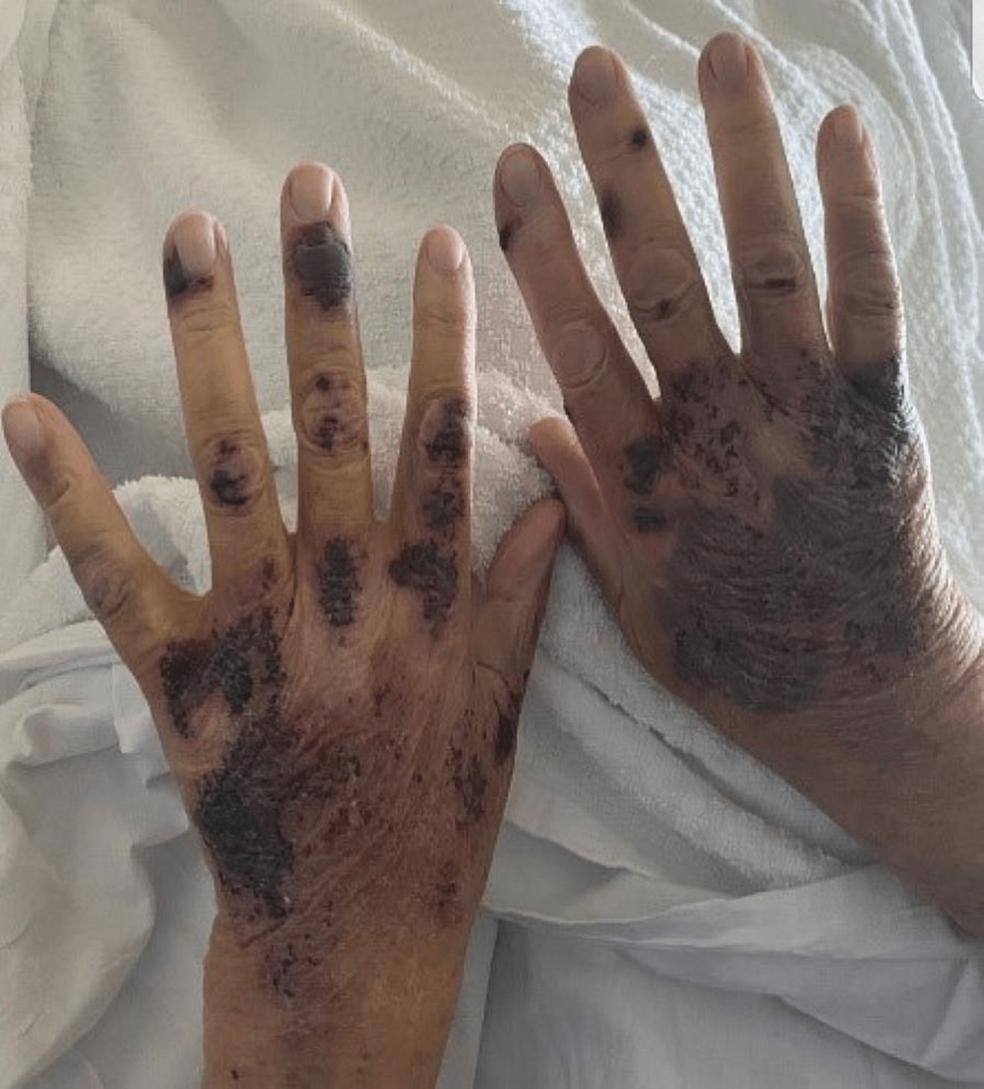
Rash on the dorsum of hands

An 84-year-old male with a history of chronic heart failure with preserved ejection fraction (HFpEF), hypertension, chronic obstructive pulmonary disease (COPD), and alcoholic liver cirrhosis presented to our hospital due to concerns about worsening lower extremity swelling and a rash. The patient had been started on spironolactone 50 mg daily by his cardiologist two weeks earlier to help with the management of hypertension and HFpEF. His other home medications included furosemide 40 mg daily, losartan 100 mg daily, and bronchodilator therapy (with inhaled umeclidinium vilanterol and inhaled fluticasone daily). On the day prior to presentation, the patient developed a painful pruritic rash on all his extremities. He had no fever, chills, diarrhea, or joint pain. All vital signs were within normal limits. Physical exam showed jugular venous distension, 3+ bilateral pitting lower extremity edema. Examination of the skin was remarkable for an erythematous, non-blanchable, purpuric rash with bullae and hyperpigmented patches on the extremities with truncal sparing and no mucosal involvement (Figures 1-2).

Laboratory evaluation showed elevated creatinine of 1.88 mg/dL (baseline creatinine level of 1.1 mg/dL), mild hyperkalemia of 5.3 mEq/L, elevated erythrocyte sedimentation rate (ESR) of 75 mm/hr, elevated C-reactive protein (CRP) of 2.5 mg/dL, normal complete blood count and coagulation panel. Urine studies demonstrated proteinuria and moderate microalbuminuria. The patient was started on intravenous furosemide for diuresis while spironolactone was discontinued in the setting of acute kidney injury and hyperkalemia.

**Figure 3:**
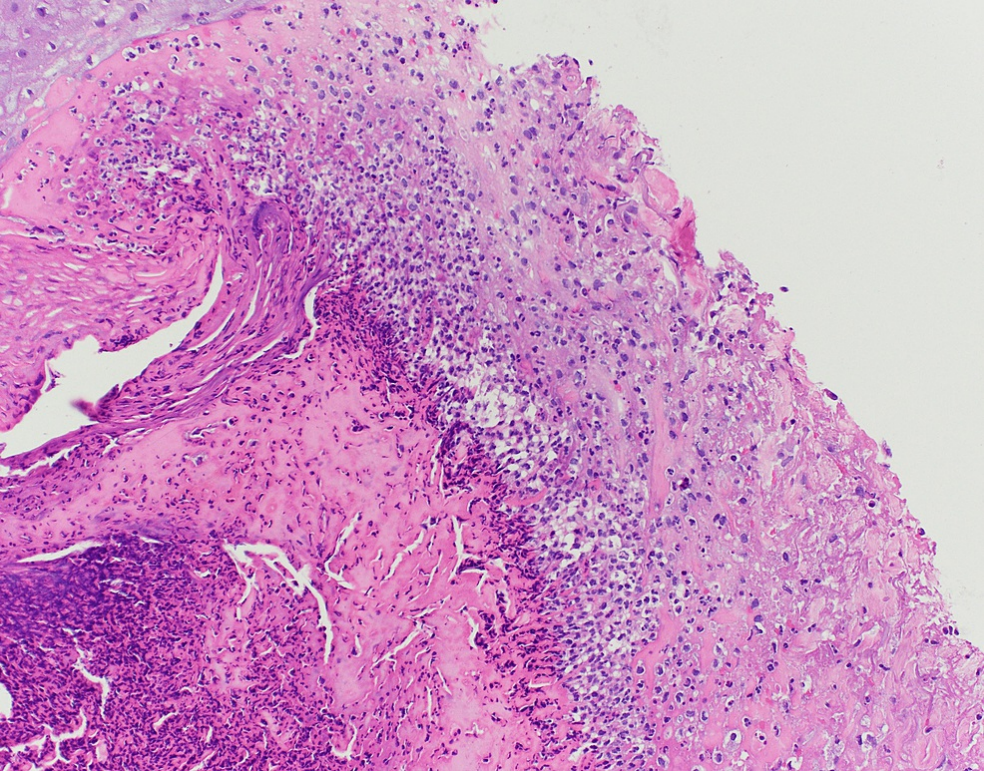
Histopathology of lesional skin The H&E stain of lesional skin demonstrates epidermal ulceration with scale crust and degenerated collagen fibers. H&E: Hematoxylin and eosin

**Figure 4:**
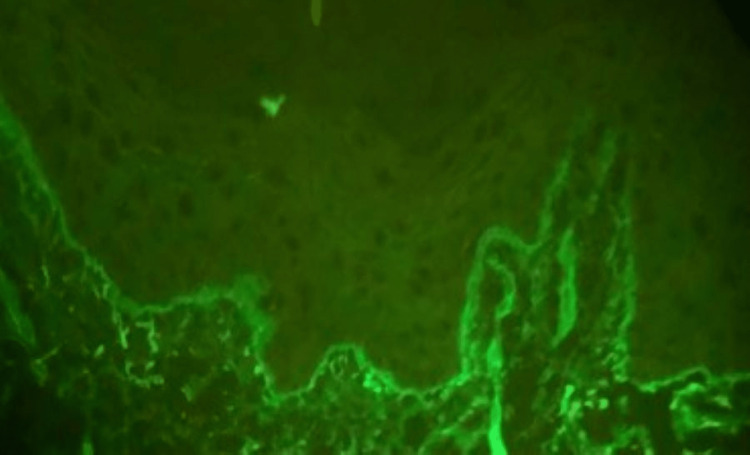
Direct immunofluorescence of perilesional skin showing linear IgA deposition at the dermal-epidermal junction

Drug-induced vasculitis was suspected due to the recent initiation of spironolactone. All serum immunological workups were negative except for a weakly positive antinuclear antibody (ANA) screen with low titer of 1:40 and cytoplasmic pattern, a weakly positive smooth muscle antibody IgG titer of 1:40, and elevated IgA level at 1033 mg/dL (normal: 68-408 mg/dL). Following the dermatology consultation, the patient was started on oral prednisone 60 mg daily with the plan to taper and discontinue over the next two to three weeks. He underwent a biopsy of lesional and perilesional skin for a definitive diagnosis. The pathology report from the lesional skin biopsy demonstrated epidermal ulceration with scale crust and degenerated collagen fibers, which are non-specific findings typically seen in blistering disease (Figure 3). Direct immunofluorescence of the perilesional skin biopsy showed linear IgA at the dermal-epidermal junction most consistent with linear IgA disease (Figure 4). At discharge, the patient was instructed to avoid spironolactone and to follow up with dermatology. His rash resolved over the next few weeks while continuing therapy with oral and topical steroids and avoiding further spironolactone use.

## Discussion

Adult-onset LABD, whether idiopathic or drug-induced, presents with subepidermal tense vesicles and bullae on erythematous inflammatory plaques or on normal skin [4,11]. Vancomycin is the most reported cause of drug-induced LABD [4,12]. Mucosal involvement occurs in up to 80% of adult patients with idiopathic LABD but mucosal surfaces are often spared in drug-induced LABD (≤40% or cases), as seen in our patient. In drug-induced LABD, lesions usually manifest within one month of drug initiation and resolve within weeks of withdrawing the offending drug, whereas spontaneous remission occurs in less than 50% of patients with idiopathic LABD [4,11,12]. Drug-induced LABD could have a more atypical and/or more severe presentation than idiopathic LABD, especially when the offending agent is not identified and discontinued early [13]. Topical or systemic corticosteroid therapy or oral dapsone is often required for treatment of idiopathic LABD and could be used to hasten recovery after discontinuation of the offending drug in patients with drug-induced LABD [4,11].

Childhood-onset LABD is typically acquired, autoimmune, and self-limited [5,14]. There have been reports of drug-induced LABD in children, usually involving antibiotics or non-steroidal anti-inflammatory agents, but drug-induced LABD has been reported more commonly in adults compared to children [15]. A typical feature of the childhood form of LABD is the presentation with annular blisters that are most prominent on the lower abdomen, thighs, and perineum; whereas this pattern of annular lesions is less common in the adult form of LABD, and lesions in adults have a predilection for the extensor surfaces of extremities, the face, and the trunk [4,14].

Due to similar presentation, there is a propensity to misdiagnose drug-induced LABD as other vesiculobullous conditions, including dermatitis herpetiformis, bullous pemphigoid, and erythema multiforme/toxic epidermal necrolysis [4,10,12], particularly when the drug involved is a previously unrecognized culprit. Other differential diagnoses include epidermolysis bullosa acquisita, prurigo nodularis, and pemphigus vulgaris. Two skin biopsies are required for definitive diagnosis [4,12]: a biopsy of the lesional skin for histology, and a second biopsy sample from the perilesional skin for direct immunofluorescence. Three criteria have been proposed for the diagnosis of LABD [5,14]: (1) vesicular or bullous skin eruptions, with or without mucosal involvement; (2) the presence of subepidermal vesicles with histologic examination demonstrating an infiltrate with neutrophilic predominance; (3) direct immunofluorescence finding of a linear pattern of IgA antibody deposited along the basement membrane zone.

The first two criteria are not specific to LABD, as they can be found in other vesiculobullous skin diseases. Although there have been reports about some degree of heterogeneity in the immunopathologic findings of LABD, the direct immunofluorescence finding is currently regarded as pathognomonic for LABD and is the most important finding required for diagnosis [5].

To qualify as a trigger of drug-induced LABD, the offending drug must have been initiated less than four weeks earlier [13]. Based on our patient’s recent medication history and clinical presentation, we suspect his LABD was due to the initiation of spironolactone two weeks earlier. To our knowledge, LABD has not previously been reported in the literature to be associated with spironolactone. Factors associated with complete remission including age ≥70 years and absence of mucosal involvement [16,17], were both fulfilled in our patient.

## Conclusions

This case highlights the importance of a complete medication review for accurate diagnosis and treatment. Elderly patients on spironolactone should be monitored closely for adverse effects and complications, including a rash. Clinicians should consider drug-induced LABD among the differential diagnoses in patients aged above 60 who present with an acute onset of vesiculobullous rash within four weeks of commencing spironolactone use. A direct immunofluorescence finding of linear IgA deposits along the basement membrane is essential for its diagnosis. Considering that spironolactone is frequently prescribed for the management of heart failure and decompensated liver cirrhosis, there is a need for heightened clinician and patient awareness of the possibility and clinical presentation of LABD associated with its use. Since this is one of the first case reports that attributes LABD to spironolactone use, there is a need for increased surveillance among patients recently exposed to spironolactone to fully elucidate the risk of LABD and establish causality.
